# Doctor-patient distancing: an early experience of telemedicine for postoperative neurosurgical care in the time of COVID-19

**DOI:** 10.1186/s41983-020-00212-0

**Published:** 2020-07-23

**Authors:** Ahmed Hamdy Ashry, Mohamed Fathalla Alsawy

**Affiliations:** 1 Department of Neurosurgery, Cairo University, Giza, Egypt; 2 Giza, Egypt

**Keywords:** COVID-19, Telemedicine, Postoperative

## Abstract

**Background:**

Telemedicine remains an important tool of healthcare services delivery especially in the era of the COVID-19 pandemic. Its advantages include saving the time and money of the patients and the prevention of infection among healthcare providers.

**Objectives:**

In this study, we aim to evaluate the effectiveness and safety of telemedicine visits in providing postoperative care of neurosurgical patients.

**Materials and methods:**

We conducted this prospective study on 30 neurosurgical patients who were evaluated after surgery via telemedicine visits for 30 days. During the virtual visits, we discussed the clinical progress of patients, wound status, treatment modification, the time of return to work, postoperative complications, and the radiological data of the patients. All the patients were given an emergency number to call in case of urgent issues. The satisfaction of patients and doctors was measured with the aid of two questionnaires.

**Results:**

The patients’ age ranged from 18 to 65 years. Twenty patients were male and 10 patients were female. We performed 20 cranial operations and 10 spinal operations. Most patients needed more than 4 h to reach the hospital. The total number of telemedicine visits was 67 visits. We received about 62 emergency calls. Pain management, seizures control, wound infection, and hydrocephalus is among issues that were evaluated and managed via telemedicine visits. The overall satisfaction rate among patients and doctors was 90% and 95%, respectively.

**Conclusion:**

Virtual outpatient clinics seem to be a safe and effective way of postoperative care especially in the time of the COVID-19 pandemic.

## Introduction

COVID-19 pandemic has put significant strains on health care systems due to lockdowns and lack of resources and protective equipment, and increases the risk of the virus transmission among patients and healthcare providers [1]. The current dilemma facing health authorities is how to provide a proper medical service not only to the patients of coronavirus but also to the patients of other diseases while preserving the lives of medical personnel [2]. Telemedicine is a way of delivering safe medical services to patients remotely via videoconferencing, sound chats, texts, and emails. Telemedicine may play a critical role in the era of the COVID-19 pandemic to decrease its impact on the economy and healthcare systems [3].

## Methods

**Fig. 1:**
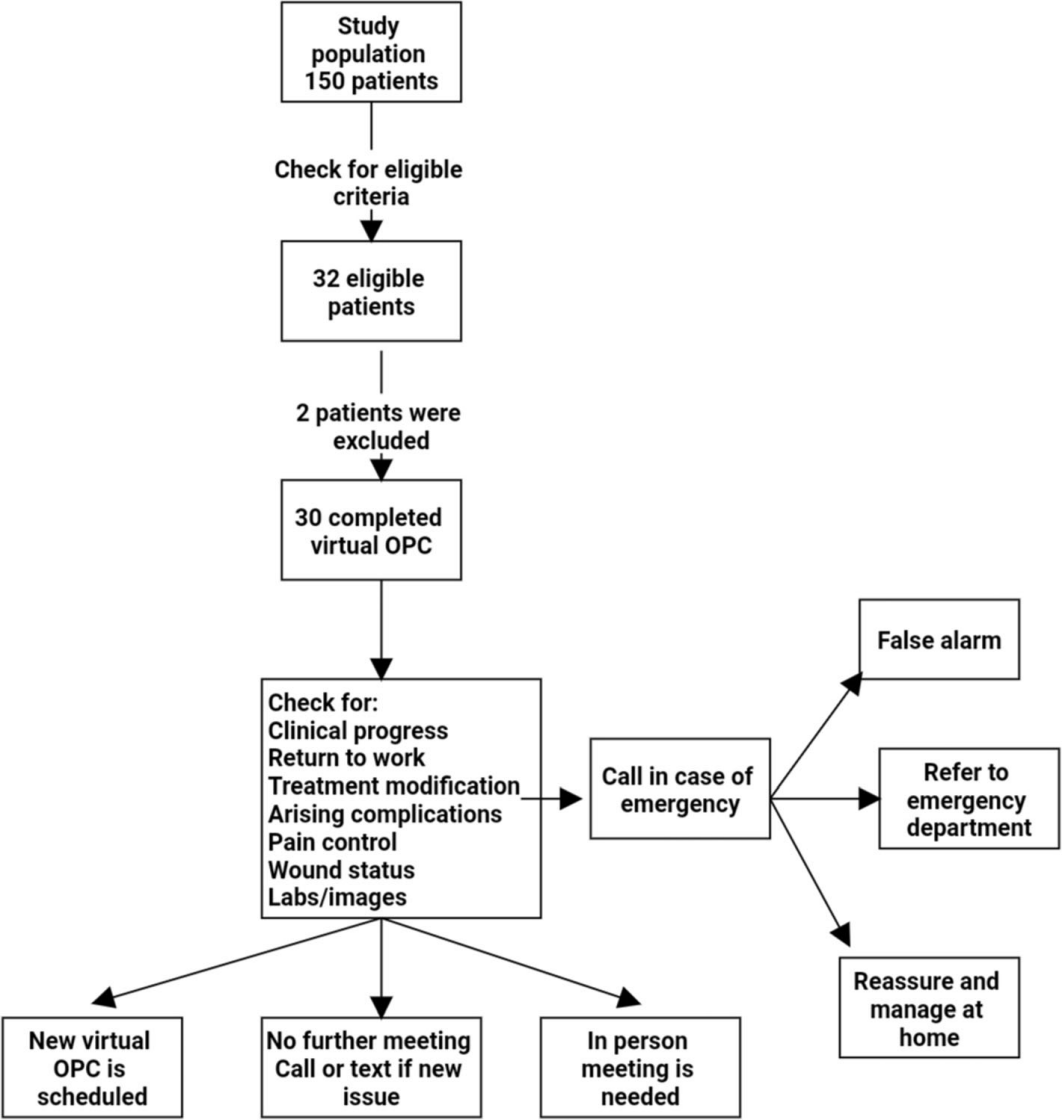
Flow chart of methodology

During March and April 2020, all the patients discharged after surgery from the Neurosurgery Department, Cairo University Hospitals, were assessed according to predetermined inclusion and exclusion criteria to check eligibility for participation in this study. We conducted this prospective study on a group of neurosurgical patients who were offered the option of postoperative evaluation via telemedicine visits for 30 days after their discharge from the hospital (Fig. 1).

We included patients above 18 years and those who agreed to participate in telemedicine visits. Patients should have smartphones or laptops and high-speed internet connections at their homes. We also included patients who experienced a smooth postoperative course in the hospital and not expected to have complications later on. We excluded patients below 18 years old and those who refused to participate in our study and preferred the traditional method of follow-up in the outpatient clinic (OPC). Patients who had old phones or lacked internet connection at their homes were excluded. We found it safer to exclude the patients who experienced a stormy postoperative course in the hospital from this early trial. Illiterate patients and those who suffered from severe visual, hearing, or cognitive dysfunction were also excluded.

If eligible for inclusion, patients were approached prior to discharge and the study rationale and potential methods of follow-up were explained. An informed written consent was obtained if they agreed to participate and exchange images, recorded videos, and medical records about their medical conditions online. We preferred to use Facebook Messenger (Facebook, Inc., Menlo Park, CA, USA) in the telemedicine visits because most patients were accustomed to it in their social life. We asked the patients to download the software application if they do not have it on their smart phones.

All the data of the patients were recorded such as age, sex, address, phone numbers, the type of surgical intervention, and how many hours the patients needed to reach the hospital.

The patients were operated upon by different consultants who treated degenerative and traumatic spinal disorders, brain tumors, hydrocephalus, traumatic head injuries, and neurovascular disorders such as brain aneurysms.

Before discharge, the patients were informed about their first appointment in the virtual outpatient clinic. They received a phone call 24 h before the determined appointment to confirm the date. They were instructed to conduct the visit in a quiet room with good lighting, to wear easily removed clothes and to have someone else to capture images of important physical findings and assist with some examination maneuvers.

Images remain a cornerstone in evaluating and diagnosing neurosurgical diseases. Images may be of poor quality if they were captured by the patient’s mobile camera. So we advised the patients to get a CD or USB copy of their images from the radiology centers. Then, they could upload their images to their mobiles or laptops and send them to the doctor by email prior to the meeting.

The virtual outpatient clinic was conducted in the presence of a consultant and a resident. The resident recorded all the recent clinical data in the patient’s progress sheet. During the telemedicine visits, we focused on the reassurance of patients about their clinical progress. We discussed the expected date of return to work or usual activities. We recorded any new symptoms such as seizures and hemiparesis. We also discussed the status of the surgical wound tenderness, collection, discharge, and the suitable time for suture removal. We assessed the patient’s pain and the need for analgesics. If necessary, we modified the treatment like tapering of steroids, discontinuation of antiepileptic drugs, or prescription of pain killers. We informed the patient about the pathology report of his tumor and his need for adjuvant therapy. We could also request new images or labs if needed.

Live communication with the patients or by recorded videos enabled the doctors to assess their mental status and speech. Assessment of the muscle strength needs hands-on examination and may be inaccurate by telemedicine. Asking the patient to do some maneuvers such as walking and holding his arms outstretched may give some information about his motor power. We could also do some remote examination with the aid of the patient’s relative.

**Fig. 2:**
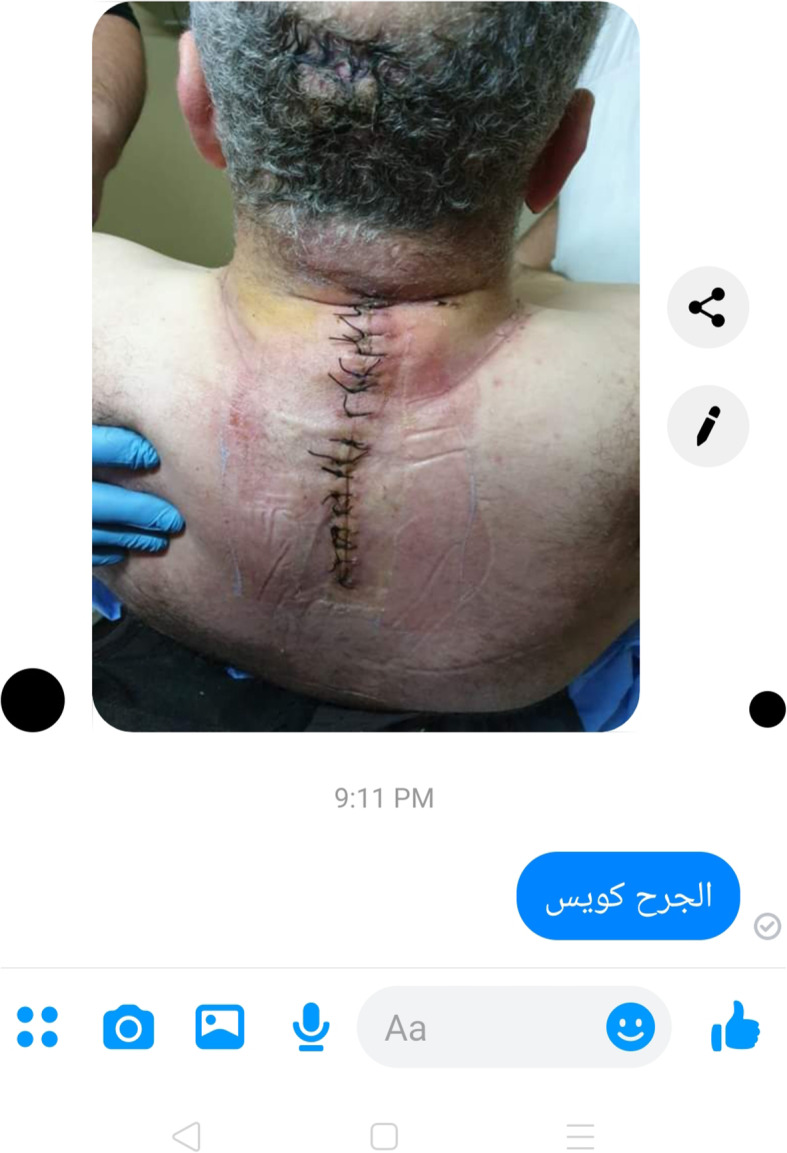
Telemedicine staff reassures a patient about his wound during online meeting

The patient could send images of his surgical wound for evaluation. If any problem was noticed in the wound, we made a decision regarding the wound either it needs surgical intervention or repeated dressing with antibiotics. If the wounds needed surgical debridement, the patient was asked to go to the hospital for readmission. If repeated dressing was decided, the patient was instructed to send daily images of his wound for frequent evaluation (Fig. 2).

At the end of the virtual meeting, one of three scenarios was expected. The doctor and patient agreed no further meeting was required, and then the patient was asked to call if he or she has any further questions or concerns. If a further virtual meeting was decided, then a new appointment was scheduled. The last scenario was when the telemedicine team felt that the patient needed an in-person visit, so an appointment at the outpatient clinic was scheduled.

All the patients were given an emergency number and they were asked to call in case of any medical emergency. The residents were responsible for answering the calls of the patients and informing the consultants about the cases if necessary. We could divide the issues discussed in the calls received from the patients into three groups. The first group included real emergency situations, and then the patients were referred to the emergency department. The second group included issues that could be monitored and managed safely at home. The last group included non-urgent issues that could be postponed to be discussed in the following telemedicine visits (false alarms).

At the end of the virtual meeting, the satisfaction of patients and doctors was evaluated according to two separate questionnaires (Tables 1 and 2)

**Table 1:** Patient satisfaction questionnaire

Questions	Yes	No
Did the staff respect your privacy?	100%	0%
Were you satisfied with audio/visual applications?	80%	20%
Did telemedicine visits save your time and money?	100%	0%
Did telemedicine visits meet your medical needs?	100%	0%
Do you think that practical sessions are necessary prior to telemedicine visits?	80%	20%
Do you prefer telemedicine visits in the future?	90%	10%
Were you overall satisfied with telemedicine visits?	90%	10%

**Table 2:** Doctor satisfaction questionnaire

Questions	Yes	No
Were you satisfied with audio/visual applications?	90%	10%
Were you satisfied with the images sent by the patients?	90%	10%
Could you perform satisfying remote examination?	80%	20%
Were you overall satisfied with telemedicine experience?	95%	5%
Do you want to expand the application of telemedicine visits after the pandemic?	90%	10%

## Results

During March and April 2020, about 150 neurosurgical patients were discharged from our department and scheduled for postoperative evaluation. All those patients were assessed according to inclusion and exclusion criteria. One hundred and eighteen patients were excluded due to different reasons. Ten patients were below 18 years old. Twelve patients did not have smartphones or laptops. Twenty patients lacked internet connection at their homes. Forty patients refused to participate in this study and preferred in-person visits. Eight patients experienced a complicated postoperative course in the hospital, and we preferred to follow-up them in the outpatient clinics. Eight patients had severe hearing, visual, or cognitive dysfunction that prevented them from participating in this study. Twenty patients were illiterate. Only 32 met our eligibility criteria, but we excluded two patients as we could not reach them after they were discharged from the hospital.

The age of the patients ranged from 18 to 65 years (mean 38.8 years). Twenty patients were male and the remaining 10 patients were females. All patients surveyed about the time they needed to reach the hospital were divided into three groups. Three patients needed less than 1 h to reach the hospital. Seven patients needed from 1 to 4 h to reach the hospital. The last group included 20 patients who needed more than 4 h to reach the hospital.

Twenty cranial surgeries were performed. They included eight operations for brain tumors excision, two operations for brain aneurysm clipping, eight operations for traumatic head injuries, and two operations for ventriculo-peritoneal shunt insertion in hydrocephalic patients. Ten spinal surgeries were performed for treatment of degenerative and traumatic spinal disorders.

The total number of telemedicine visits was 67 visits (average 2.23 visits for each patient). After the first telemedicine visit, three patients did not need further meetings and they were asked to call in case of any arising issues. The remaining patients were scheduled for further telemedicine visits. During the postoperative period of follow-up, only two patients needed in-person visits, and they were referred to the hospital for readmission. One of them had wound infection, and he was sent to the hospital for surgical debridement after the failure of conservative measures at home. The other patient was scheduled to have ventriculo-peritoneal shunt insertion for hydrocephalus after brain tumor excision.

The total number of emergency calls was 32 calls (average 1.06 calls from each patient). Only two patients called in case of real emergency situations, and they were referred to the emergency department. One of the patients experienced disturbed conscious level after brain tumor excision and the other patient suffered from status epilepticus. We received 10 calls from patients while they intended to go to the hospital for potential readmission. Out staff reassured the patients that their issues could be monitored and managed at home and could prevent unnecessary visits. These issues included the appearance of subcutaneous collection at the site of the surgical incision (three calls), postoperative fever (two calls), and uncontrolled pain (five calls).

We received 20 calls about non-urgent issues that could be postponed to be discussed in the following telemedicine visits. These calls were about the intake of certain types of foods and drinks (10 calls), performing some daily activities such as driving and climbing stairs (five calls), the proper time of sexual intercourse after surgery (two calls), and the proper time of discontinuation of the external lumbar brace or cervical neck collar (three calls).

All the complications and arising issues that appeared in the postoperative period and evaluated via telemedicine visits were recorded. Ten patients suffered from uncontrolled pain, and they were managed by prescription of pain killers. Two patients suffered from repeated attacks of focal fits which could be controlled by modification of their antiepileptic drugs. Three patients experienced postoperative wound infection. They were asked to send daily images of their wounds for frequent evaluation. Two patients were treated successfully at home with repeated dressings and antibiotics. The remaining patient was readmitted at the hospital for surgical debridement after the failure of conservative measures at home. One patient complained of manifestations of increased intracranial pressure after brain tumor excision. He was asked to do a CT brain (using a 16 multi-slice CT scanner, Toshiba Alexion, Japan) which revealed hydrocephalus, and he was referred to the hospital for ventriculo-peritoneal shunt insertion.

After telemedicine visits, all the patients were questioned about their satisfaction with the processes and outcomes of the experience. Ninety percent of patients were overall satisfied with telemedicine visits which met all their medical needs. All the patients reported that the telemedicine staff respected their privacy and dignity. Eighty percent of patients were satisfied with the quality of sound and video transmission, while 20% of patients were not satisfied due to poor internet connections in some visits. All the patients reported that telemedicine visits saved their time and travel expenses. Ninety percent of patients preferred telemedicine visits in the future. Eighty percent of patients suggested that performing a simulation session before discharge from the hospital is necessary.

Ten residents and 10 consultants participated in this study with an overall satisfaction rate of 95%. Only 20% of doctors reported difficulties in performing remote clinical evaluation especially muscle strength examination in some spinal cases. Ninety percent of doctors reported that the images sent by the patients were helpful while the remaining doctors were not satisfied and they asked their patients to recapture the images of their wounds and radiological data for better evaluation. Ninety percent of doctors were satisfied with the quality of sound and video transmission during telemedicine visits. Ninety percent of doctors preferred to expand the application of telemedicine experience after the pandemic (Table 3).

**Table 3:** Summary of results

	Number of patients
Age	
18–30 years	10
30–50 years	15
50–65 years	5
Sex	
Male	20
Female	10
Time needed to reach the hospital	
Less than 1 h	3
1–4 h	7
More than 4 h	20
Type of operation	
Cranial	20
Brain tumor excision	8
Traumatic brain injuries	8
Aneurysm clipping	2
Hydrocephalus	2
Spinal	10
Number of telemedicine visits	67
Number of emergency calls	32
Real emergency situations	2
Disturbed consciousness	1
Status epilepticus	1
Home management	10
Wound collection	3
Postoperative fever	2
Uncontrolled pain	5
False alarms	20
Intake of certain types of food	10
Time of sexual intercourse	2
Performing some activities	5
Discontinuation of a brace	3
Evaluated issues during virtual visits	
Wound infection	3 (2 conservative/1 surgical debridement)
Uncontrolled pain	10 (prescription of pain killers)
Focal seizures	2 (modification of antiepileptic drugs)
Hydrocephalus	1 (readmitted for shunt insertion)
Number of readmissions	2 (wound infection/hydrocephalus)
Number of emergency referral	2 (disturbed consciousness/status epilepticus)

## Discussion

COVID-19 pandemic has put great pressures on healthcare systems worldwide and imposed many strict precautions such as isolation, quarantine, and physical distancing [1].

In Egypt, many hospitals, even in the private sector, have been designated for isolation and treatment of COVID-19 patients. The Egyptian ministry of health made great efforts to raise the efficiency of isolation hospitals and provide resources and protective equipment. Being dependent for long decades on donations and self-efforts, the ability of these hospitals to accommodate this flood of COVID-19 patients raises many concerns especially in a community of 100 million people. The public health system in Egypt suffers from severe shortage of physicians due to their emigration searching for better working circumstances. This shortage was exacerbated by the increasing numbers of coronavirus infections among the healthcare providers raising a red flag amid the crisis.

Other hospitals face major challenges to provide appropriate medical service to the patients of other diseases while adhering to the strict rules of protection and infection control. All non-urgent surgeries and unnecessary visits were postponed to preserve resources and decrease the virus transmission among the patients and medical staff [4]. All these challenges facing our healthcare system forced us to think about unconventional methods of providing medical care. In spite of being the standard practice in neurosurgery, face-to-face visits seem to be risky [5].

In the developed countries, COVID-19 pandemic has accelerated the adoption of telemedicine services in their healthcare systems [6]. In Egypt telemedicine projects face a lot of difficulties with very little publications about applied experiences [7]. Nevertheless, we tried to implement an early experience of telemedicine visits by the establishment of virtual outpatient clinics for the evaluation of neurosurgical patients after surgery. Literature about telemedicine in the field of neurosurgery are very scarce and limited to triaging of patients of neurotrauma [8], postoperative care of patients after elective surgeries [9], and evaluation of socioeconomic benefits for patients in remote areas [10].

In Egypt, many doctors and nurses use smartphones in their daily activities. They asked the patients to send the images of their wounds and radiological data for consultation. In spite of being a useful tool, it lacks the security standards and carries the risk of disclosure of confidential data of the patients [11].

The advantages of telemedicine projects are too obvious. They could save time and travel expense for the patients [9]. Kasr Alainy Hospital is a big tertiary center which serves thousands of patients in rural and remote areas. In our study, most of the patients needed more than 4 h to reach the hospital. Traveling for a long time on busy transportation carries high risk of infection among the patients. Many patients would have to spend the night in Cairo due to the curfew imposed in response to COVID-19 pandemic. Telemedicine visits made the patients avoid long-distance travel, helped reduce rates of infection, and saved money especially in unfavorable economic conditions imposed by the pandemic.

Communication with the patients by phone helped us prevent unnecessary visits, detect complications early, and respond quickly to emergencies. Unfortunately, we received many calls about non-urgent issues that could be discussed later via telemedicine visits. This could be overcome by educating the patients about the possible complications they might experience and giving them a written checklist of warning symptoms. The ability of the patients to call the doctors at any time leads to the feeling of intrusion and increases the level of dissatisfaction among healthcare providers [12].

The telemedicine projects face a lot of difficulties in developing countries. Lack of proper funding and infrastructures is one of these obstacles [13]. Implementation of telemedicine projects requires the provision of desktop computers and high-speed internet connection in hospitals. In our study, we relied on simpler technology represented by smartphones connected to the internet through cellular data or Wi-Fi. Despite some connection problems we faced in our study, this method enabled us to work from home and decrease the spread of infection among the patients and doctors.

The high rate of illiteracy and ignorance of technology in Egypt stands as a stumbling block in the way of implementation of telemedicine projects [7]. Poor internet connections and sophisticated web-based applications make using telemedicine services confusing and boring. Most doctors and patients were familiar with the application used in our study. They use Facebook Messenger (Facebook, Inc., Menlo Park, CA, USA) daily to communicate with friends and families in their social life. In the coming times, we prefer to do simulation sessions in the hospital before the discharge of the patients to make sure that they can use the application easily.

Most of the doctors are accustomed to classic ways of diagnosis, treatment, and follow-up which increases the resistance to telemedicine project implementation. Egyptian culture and traditions impede the patients’ acceptance of treatment without in-person visits [7]. In our study, we found that a large percentage of those who refused telemedicine visits were female patients. We believe that female patients may feel embarrassed during online consultations due to the sense of lack of privacy. This can be overcome by publishing programs to increase the awareness of the patients and healthcare providers about the importance of telemedicine visits in the various media outlets.

One of the disadvantages that we faced in our study is the inability of some surgeons to perform accurate remote clinical examinations especially motor power assessment in some spinal cases. In the future, we might support modern technologies which would enable us to perform satisfying remote examination [14].

In our study, we reported high overall satisfaction with telemedicine visits among patients and doctors. The COVID-19 pandemic may have bright aspects that could force the officials to make a surge in telemedicine technology to serve healthcare processes including diagnosis, treatment, follow-up, infection control, and medical research.

## Conclusion

COVID-19 pandemic has a disastrous impact on various fields of life all over the world. Healthcare systems have become overwhelmed with massive numbers of infected cases which decreased their efficiency in facing the pandemic. Overcrowded outpatient clinics remain a major cause of infection spread among patients and healthcare providers. This enforced us to consider an alternative way other than the standard clinical practice to deliver adequate medical service to our patients. The establishment of virtual outpatient clinics for postoperative care of neurosurgical patients seems to be effective and safe. We found that most complications and concerns that may arise in the postoperative period could be managed safely via virtual ways. This study is an excellent starting point, but more perseverance and enthusiasm is needed to improve the results and overcome the obstacles.

## Data Availability

The dataset used and analyzed during the current study are available from the corresponding author on reasonable request.

## Abbreviations

COVID-19: Coronavirus disease 2019
CD: Compact disc
USB: Universal serial bus
CT: Computerized tomography
OPC: Outpatient clinic
